# Biosynthetic Potentials of Metabolites and Their Hierarchical Organization

**DOI:** 10.1371/journal.pcbi.1000049

**Published:** 2008-04-04

**Authors:** Franziska Matthäus, Carlos Salazar, Oliver Ebenhöh

**Affiliations:** 1 Interdisciplinary Center for Scientific Computing, University of Heidelberg, Heidelberg, Germany; 2 German Cancer Research Center, Heidelberg, Germany; 3 Max-Planck-Institute for Molecular Plant Physiology, Potsdam-Golm, Germany; 4 Institute for Biochemistry and Biology, University of Potsdam, Potsdam, Germany; University of Washington, United States of America

## Abstract

A major challenge in systems biology is to understand how complex and highly connected metabolic networks are organized. The structure of these networks is investigated here by identifying sets of metabolites that have a similar biosynthetic potential. We measure the biosynthetic potential of a particular compound by determining all metabolites than can be produced from it and, following a terminology introduced previously, call this set the scope of the compound. To identify groups of compounds with similar scopes, we apply a hierarchical clustering method. We find that compounds within the same cluster often display similar chemical structures and appear in the same metabolic pathway. For each cluster we define a consensus scope by determining a set of metabolites that is most similar to all scopes within the cluster. This allows for a generalization from scopes of single compounds to scopes of a chemical family. We observe that most of the resulting consensus scopes overlap or are fully contained in others, revealing a hierarchical ordering of metabolites according to their biosynthetic potential. Our investigations show that this hierarchy is not only determined by the chemical complexity of the metabolites, but also strongly by their biological function. As a general tendency, metabolites which are necessary for essential cellular processes exhibit a larger biosynthetic potential than those involved in secondary metabolism. A central result is that chemically very similar substances with different biological functions may differ significantly in their biosynthetic potentials. Our studies provide an important step towards understanding fundamental design principles of metabolic networks determined by the structural and functional complexity of metabolites.

## Author Summary

Life is based on the ability of cells to convert raw materials into complex chemicals like proteins or DNA. This ability is obtained through the interplay of a large number of enzymes, which are specialized proteins, each facilitating one specific chemical transformation. Since the products of one reaction can again be substrates for others, the entirety of all reactions forms a large and complex network in which important substances can be produced from many different combinations of simple chemicals and through a variety of pathways. The aim of our work is to gain understanding of the structural design of these networks and the evolutionary principles shaping them. We propose a computational strategy which allows us to pinpoint characteristic structural and functional properties distinguishing networks characterizing living processes from those that may occur in inanimate matter. Our approach reveals an intricate and unexpected hierarchical organization of the network, and gives rise to new hypotheses regarding the evolutionary origins of metabolism.

## Introduction

Cellular metabolism is mediated by highly efficient and specialized enzymes catalyzing chemical transformations of substrates into products. Since the products of a particular reaction may serve as substrates for other reactions, the entirety of the biochemical reactions forms a complex and highly connected metabolic network. With the sequencing of whole genomes of an ever increasing number of organisms and the emergence of biochemical databases such as KEGG [1], Brenda [2] or MetaCyc [3], which are based on genomic information, large-scale metabolic networks have become accessible. The KEGG database, for example, holds biochemical reactions of several hundred organisms, forming a metabolic network with over 6000 reactions connecting over 5000 metabolites. Whereas the wiring principles of small metabolic systems such as single biochemical pathways or a small number of interacting pathways are generally easily comprehensible, elucidating the organization of large-scale metabolic networks still poses a major challenge in the field of systems biology.

While a network of 6000 reactions is large in the sense that it is computationally challenging, this number represents only a tiny fraction of all theoretically possible, chemically feasible reactions. So why did enzymes evolve for these reactions but not for others? And what were the selective pressures that lead to this particular selection? While we are still far from answering these intriguing questions satisfactorily, it is plausible to assume that the selection was not random but a result of a long evolutionary process which must have left its imprint in the structure of the contemporary metabolic network. We use this assumption as our working hypothesis and identify an interesting hierarchical organization which seems to be an intrinsic property of metabolism and robust against moderate changes in network structure and other specific assumptions like the availability of particular chemicals. Our results inspire some speculations on the above raised questions and we outline some possible continuations of this work with the aim to get further insight into the principles that guided metabolic evolution.

Several approaches to analyze the structure of large-scale metabolic networks have emerged in recent years. Graph theoretical approaches have revealed characteristic global features. It has been shown that metabolic networks exhibit a small world character [4], possess a scale-free topology [5] and display a hierarchical organization [6]. However, all these approaches rely on a representation of a metabolic network as a graph. There are many alternative ways to construct a graph from a metabolic network (see for example [7]). A characteristic of most of the applied approaches is that it is in general not possible to reconstruct the original metabolic network from the graph, since in the simplification process important biochemical information is lost. Moreover, graph theoretical results may strongly depend on the particular representation. For example, the small world property has been shown for a graph, in which the nodes represent metabolites connected by an edge if they participate together in a biochemical reaction. If, however, metabolites are only connected by an edge if there exists a reaction that transfers at least one carbon atom from one metabolite to the other, the small world property is lost completely [8].

The concepts of flux balance analysis [9], elementary flux modes [10] or extreme pathways [11] all aim at characterizing the possible flux distributions through the biochemical reactions when certain external metabolites are either provided by the environment or can be released into extracellular medium. Such an approach is well suited for the investigation of metabolic networks of selected organisms for which the fluxes of metabolites over the cellular membrane have been well characterized, so that it is clear which biochemical compounds have to be considered as external. Based on flux balance analysis, it has been shown that experimentally measured flux distributions in *E. coli* correspond well to distributions calculated under the premise that biomass production is maximized [12].

For the analysis of the network comprising the entirety of all biochemical reactions, it is impossible to decide which metabolites should be considered as external, since the role may differ greatly among different organisms or within cells of different tissues. A novel strategy for the analysis of large-scale metabolic network, that is less dependent on the knowledge which particular metabolites are external, has recently been proposed. The so-called method of network expansion [13],[14] is based on the basic biochemical fact that only those reactions may take place which use the available substrates and that the products of these reactions may in turn be utilized by other reactions. With a number of given substrates (the seed), a series of metabolic networks is constructed, where in each step the network is expanded by those reactions that utilize only the seed and those metabolites which are products of reactions incorporated in previous steps. The set of metabolites within the final network is called the scope of the seed and, by construction, comprises all substances that the network may produce when only the seed compounds are available as external resources. The scope describes the biosynthetic potential carried by the seed compounds and thus in a natural way links structural and functional properties of metabolic networks.

In the present work, we aim at elucidating the global organization of functional aspects of metabolism by comparing the biosynthetic potentials of the different metabolites. For this, we extend studies carried out by us previously [15]. There, we observed that many compounds exhibit very similar potentials and introduced the notion of a *consensus scope*, characterizing the biosynthetic potential of a large group of metabolites. Whereas in our previous studies [15] we focused on the technical aspects and compared different dimensionality reduction methods, we concentrate in this work on the generalization of our results and in particular on their interpretation in a biological and evolutionary context.

We find that many compounds can be grouped into biologically meaningful clusters, displaying a typical biosynthetic potential. We demonstrate that these typical potentials also characterize the combined potential of sets of metabolites. Furthermore, we observe that a similar biosynthetic potential of metabolites can often be connected with common chemical properties. However, in some cases chemically similar substances may exhibit dramatically different biosynthetic potentials and, moreover, clearly distinct biological functions may be assigned to such metabolites.

The paper is organized as follows: The Results section consists of three parts in which we describe i) the results from the hierarchical clustering as well as the construction of consensus scopes, ii) the chemical properties of compounds belonging to the same cluster, and the hierarchical organization of the biosynthetic potentials, and iii) the generalization to combined biosynthetic potentials of sets of metabolites. For readability, some results and definitions from [15] have been included in the first two parts of that section. In the Discussion section, our results are discussed. And finally, in the Methods section, details about the applied calculations are provided.

## Results

### Clustering Metabolites by Their Biosynthetic Potential

The aim of this work is to identify organizational principles in the metabolic network which is spanned by the entirety of biochemical reactions. For our analysis, we have retrieved enzymatic reactions from over 200 organisms from the KEGG database [1]. After curation of this information (see Methods), the network consists of 4811 reactions involving a total of 4104 metabolites. We characterize all biochemical compounds by their biosynthetic potential.

#### Definition of the biosynthetic potential (scope) of a metabolite

By the biosynthetic potential of a particular metabolite we understand the set of all metabolites which can in principle be synthesized by all available enzymatic reactions when exclusively the metabolite itself, water and oxygen are available as substrates. This quantity is determined using the network expansion algorithm as described in [14] and, following their terminology, will be called the scope of the compound.

A characteristic known from previous studies [14],[16] is that most metabolites carry a small biosynthetic potential. In fact, for almost three quarters (3027) of the metabolites, the potential is so low that they allow for the production of less than 8 new compounds. Also in agreement with previous results [15], we find that several compounds carry exactly the same biosynthetic potential. Moreover, it is often the case that compounds possess very similar biosynthetic potentials, meaning that many metabolites may be produced from either compound, but the synthesis of a small number of metabolites requires a specific starting compound.

Inspired by this observation, we investigate whether metabolites may be grouped into biologically meaningful classes characterized by typical biosynthetic potentials. For this, we introduce a distance measure reflecting the dissimilarities of the biosynthetic potentials of two compounds. Such a measure should be small if a similar set of metabolites may be produced from either of the two compounds, and large if these sets are different, irrespective of the total number of metabolites that may be synthesized. A distance measure fulfilling this condition is based on the Jaccard coefficient. For two sets *A* and *B*, this coefficient is given as the ratio between the number of elements contained in both sets, 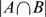, and the number of elements appearing in at least one of the sets, 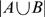. If we denote by *S_i_* and *S_j_* the sets of metabolites defined by the scopes of two compounds *i* and *j*, respectively, we characterize the dissimilarity of the biosynthetic potential of the two compounds by the distance
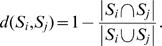
(1)By construction all scopes have five compounds in common, namely those contained in the scope of water and oxygen (in addition to the two seed compounds, the scope also includes H_2_O_2_, H^+^, and the dioxygen radical O_2_^−^). We remove these compounds from all sets *S_i_* for the calculation of the distances. In this way, *d*(*S_i_*,*S_j_*) is zero if the biosynthetic potentials are identical, and one if they do not have a single metabolite in common.

Based on these dissimilarities, we perform a hierarchical clustering (see Methods) to identify clusters of compounds carrying a similar biosynthetic potential. For a merging distance of 0.2, we find 12 clusters with at least 10 elements (see Dataset S1 in the supporting information for a detailed list), called cluster I to cluster XII. Apart from these, 2433 metabolites are not assigned to any cluster. The remaining 894 metabolites are assigned to clusters with less than 10 elements. A closer inspection reveals that most of the metabolites which have not been assigned to a cluster or have been assigned to a very small cluster belong to the large group of metabolites with a low biosynthetic potential. There exists, however, one exceptional case among the large number of very small clusters. This cluster contains four metabolites (APS, PAPS, Dephospho-CoA, UDP-6-sulfoquinovose) exhibiting the largest, identical, biosynthetic potential, allowing for the production of 2178 metabolites. To account for the outstanding role of these four metabolites, we also consider this cluster in our detailed analysis and denote it by the label XIII.

The quality of the clustering is assessed by a parameter *β* quantifying the ratio between the cluster radius and the cluster separation (see Methods). This value is small (<0.5) for all clusters I-XIII (see Table 1), assuring that the clusters are well separated and the assignment of metabolites to clusters is unambiguous.

**Table 1:** Clusters of biochemical compounds determined by a hierarchical clustering algorithm.

Label	Cluster Elements and Representative	s_clust_	s_cons_	*β*
I	Organic compounds containing nitrogen: amino acids, nucleosides, nucleobases, amino acid derivatives (L-Glutamate)	261	423	0.33
II	Organic compounds not containing nitrogen: ketoacids, diols, di- and tricarboxylic acids, hydroxyacids (Pyruvate)	183	148	0.47
III	Compounds with heterocyclic bases, sugars and phosphate groups: nucleotides, deoxynucleotides, sugar nucleotides, cofactors, nucleotide precursors, nucleoside derivatives, amino acid derivatives, glycolipids (ATP)	102	1549	0.09
IV	Sugar phosphates, phospholipids and inositolphosphates (D-Fructose 6-phosphate)	57	109	0.23
V	Sugars, glycosides (D-Glucose)	41	31	0.19
VI	Deoxynucleotides and their sugars with thymine as a base, sugar phosphates (dTDP)	34	305	0.14
VII	Eicosanoids (Arachidonate)	23	23	0
VIII	CO, CO2, dicarboxylic acids, ketoacids, hydroxyacids, fatty acids, amino acids, flavonoids (Glyoxylate)	22	12	0.15
IX	Coenzyme A compounds (Acetyl-CoA)	19	203	0.1
X	Activated forms of terpenes and terpenoids (Isopentenyldiphosphate)	13	49	0
XI	Indole alkaloids (Strictosidineaglycone)	12	11	0.16
XII	Aromatic organic compounds with a benzene ring (Quinone)	10	9	0.19
XIII	Nucleotide sulfur compounds (Adenosinephosphosulfate)	4	2178	0

For each cluster, we list structural categories characterizing the majority of the cluster members, and a cluster representative metabolite whose scope is identical to the consensus scope. Furthermore, the cluster size s_clust_, the consensus scope size s_cons_ as well as the parameter *β* measuring quality of the clustering is given.

The observation that biosynthetically potent compounds form clearly distinguishable groups, characterized by a similar synthesizing potential, suggests that the number of significantly different scopes is very small. Even though scopes of different compounds are rarely completely identical, every scope is at least *similar* to one of a small set of typical scopes. These thoughts lead to the following generalization of the notion of the scope of a compound [15].

#### Definition of the consensus scope of a cluster

For a cluster of compounds with similar biosynthetic potential, we define the *consensus scope* as the set of metabolites which appear in the majority of all scopes in the cluster.

Consensus scopes provide a compact characterization of complex metabolic networks. They define a small set of typical biosynthetic potentials with the property that the scope of any compound is either very small or similar to exactly one of these.

In Table 1, we give a summary of the thirteen identified clusters. The compounds within a cluster are characterized based on chemical properties. The number of compounds (cluster sizes) and the size of their consensus scope are given, as well as the parameter *β* describing the cluster separation. Additionally, a cluster representative is given in parenthesis behind the chemical characterization of the compounds. These representatives possess a scope identical to the cluster's consensus scope. Interestingly, for every cluster such a representative exists even though the definition of the consensus scope does not guarantee that it actually represents a valid scope of one or several metabolites. If, for example, consensus scopes were calculated not for clusters of compounds with similar biosynthetic potential but for arbitrary sets, the result will in general not correspond to a scope of a single metabolite. Rather, the concept of consensus scopes only makes sense in conjunction with compound clusters. And the observation that all clusters possess representatives confirms the high quality of the clusters.

Clusters I to XIII contain together 781 compounds. Apart from these there are 3027 compounds with a very low biosynthetic potential of less than 8 new compounds. The remaining 296 metabolites are distributed among 70 small clusters with less than 10 members. We do not include these in our detailed analysis. The sizes of the consensus scopes of the clusters I-XIII range from 9 to 2178. Clearly, the consensus scope size is not correlated with the cluster size; it can be smaller or larger. In the cases where the consensus scope is larger than the cluster size, the majority of metabolites within the cluster are also found in the consensus scope. This property is not a direct consequence of the definition of the consensus scopes, it rather demonstrates that the majority of metabolites in such clusters are interconvertible, meaning they are mutually contained in each other's scope. If the consensus scope is smaller than the cluster size, there exist necessarily compounds within the cluster which are not contained in the consensus scope. This characteristic of consensus scopes is fundamentally different from the original notion of the scope of a compound, in which any compound must by definition be included in its own scope.

In the definition of the consensus scope, an arbitrary threshold value of 50% was introduced. To verify that the definition is robust against variations of this value, we varied the threshold between 30 and 70% and found that the consensus scopes remain exactly identical. The only exception is cluster VI, which consists of two subclusters of similar size, one having a consensus scope of size 283, and the other of size 305. Because the two subclusters are of similar size, the smaller consensus scope will be obtained if a higher threshold value than 50% is chosen.

### The Hierarchies of Biosynthetic Potentials

**Figure 1:**
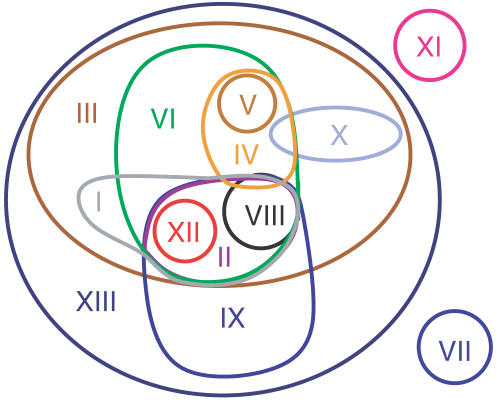
Consensus scope overlap for the 13 clusters obtained with the hierarchical clustering method. Two of the consensus scopes (VII, XI) are mutually disjoint, while others overlap (e.g., III and IX), and some consensus scopes are fully contained in others (e.g., VI in III).

The extreme variation in consensus scope sizes raises the question whether they may be ordered by increasing biosynthetic potential. In fact, some consensus scopes are contained in others, some are mutually disjoint and others partially overlap. We schematically visualize the pairwise overlaps in Figure 1. The figure shows that the immensely complex metabolic network displays an intricate hierarchical organization with respect to the biosynthetic potentials of the participating compounds. In the following, we will analyze similarities and differences in the chemical structure of metabolites belonging to the same cluster and particularly address the question whether the identified hierarchy may be explained by chemical structure alone or whether the biological role of metabolites or clusters of metabolites is also reflected in the metabolic organization.

The largest consensus scope is formed by the four compounds in cluster XIII. It is identical to the scope of adenylyl sulfate (APS) and contains as subsets all other consensus scopes except those of clusters VII and XI. The second largest consensus scope is reached by metabolites of cluster III. Eight of the remaining consensus scopes are subsets thereof. This cluster contains organic compounds consisting of heterocyclic bases, sugars and phosphate groups, for example nucleotides, deoxynucleotides (except those with thymine as base), nucleotide sugars, coenzymes except coenzyme A, and second messengers such as cAMP and other nucleotide derivatives. Many compounds contained in the cluster, such as ATP or NADH, are involved in energy metabolism. They are necessary for typical metabolic reactions, such as phosphate group transfer and redox reactions. The consensus scope of cluster III is identical to the scope of ATP. Cluster VI has the largest consensus scope completely contained in the scope of ATP. The cluster consists predominantly of those deoxynucleotides and deoxynucleotide sugars with thymine as their base. Apparently, their biosynthetic potential is smaller than that of other deoxynucleotides. This is surprising in view of the fact that dUTP, a member of cluster III, and dTTP, a member of cluster VI, show very similar chemical structures. However, even though dTTP is slightly more complex than dUTP because it possesses an additional methyl group, its biosynthetic capacity is much lower. While 1549 compounds may be synthesized from dUTP, dTTP allows for the production of only 305 compounds. This finding demonstrates that the chemical complexity of a biochemical compound is not the only determinant for the biosynthetic potential it carries.

The consensus scope of cluster IV, consisting mainly of sugar phosphates, is completely contained in the consensus scope of cluster VI. The reduced biosynthetic potential is easily explained by the fact that sugar phosphates appear as chemical subunits in larger metabolites contained in clusters III and VI. However, sugar phosphates do not contain nitrogen, therefore, from them alone, e.g. nucleotides cannot be produced. Sugars form cluster V. Obviously, since the phosphate group is not available, their biosynthetic potential is even smaller, and consequently the consensus scope is completely contained in the consensus scope of cluster IV. Most other inclusion relations can also be explained by the presence or absence of characteristic chemical groups.

Cluster II consists of organic acids not containing nitrogen. Its consensus scope, identical to the scope of pyruvate, is completely contained in that of cluster VI, but only shows a small overlap with that of cluster IV. It completely contains the consensus scopes of clusters VIII and XII. The composition of cluster VIII is rather diverse, ranging from small molecules such as glyoxylate to relatively large secondary metabolites including polyketides and flavonoids. A common property of these metabolites is that they can be oxidized to CO, CO_2_ or small carboxylic acids. These products also form the small consensus scope (size 12) of the cluster. Metabolites within cluster XII share the common feature that they contain an aromatic 6-carbon ring. Its small consensus scope (size 14) is almost identical to the cluster itself.

Interestingly, there are two clusters (VII and XI), whose consensus scopes do not overlap with other consensus scopes. Metabolites within cluster VII are all derived from 20-carbon polyunsaturated essential fatty acids, known as eicosanoids. These are highly specialized compounds functioning as signaling molecules in mammals during inflammation and immune response [17]. All metabolites in the cluster possess identical scopes (cluster radius zero, see Table 1), indicating that only a very special group of chemicals can be produced from them and conversely, those chemicals can exclusively be produced from eicosanoids. It is intriguing that structural considerations alone reveal such a clear separation of this cluster from the rest of the metabolism, reflecting the specialized role of eicosanoid metabolism.

Cluster XI represents a group of nitrogen heterocyclic compounds with the common feature that all contain an indol group. All of these compounds are involved in the indole and ipecac alkaloid biosynthesis pathways. Again, it is striking that the purely structural approach reveals a separate cluster, consisting of metabolites that play a highly specialized role in metabolism. Similarly to the eicosanoids mentioned above, indole alkaloids function as signaling molecules; however, they are predominantly present in plants.

**Figure 2:**
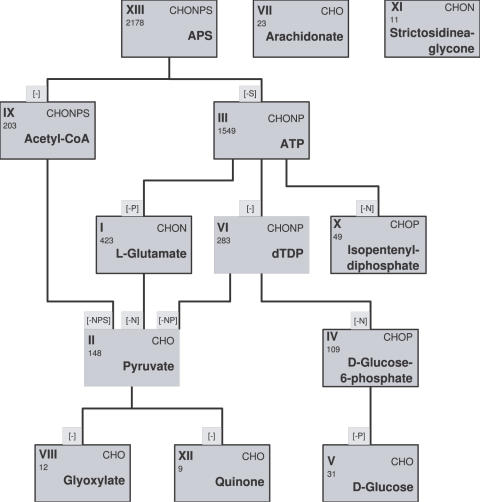
Hierarchy of compounds based on their biosynthetic potentials. Each box denotes a distinct consensus scope. On the top-left corner of each box, the cluster label and consensus scope size are shown. On the top-right corner, the chemical elements present in most cluster metabolites are given. Further, a representative metabolite of the cluster, whose scope is identical to the cluster's consensus scope, is given. Two consensus scopes are connected by an edge if the lower one is completely contained in the upper one. If the inclusion can be explained by differences in the chemical elements within the cluster members, the missing elements have been noted at the corresponding edge.

In Figure 2, the hierarchical ordering of the consensus scopes is displayed in a tree form. The boxes contain a cluster representative (a compound with a scope identical to the consensus scope), the cluster label and the consensus scope size, as well as the chemical elements present in most metabolites of the corresponding cluster. In the drawing, clusters with a large biosynthetic potential are positioned above clusters with a lower biosynthetic potential. A line between two clusters is drawn if the consensus scope of the cluster positioned below is a subset of the consensus scope of the cluster positioned above. For clarity, indirect inclusions are not depicted (although the scope of glucose is naturally contained in that of APS, a line has not been drawn). Also partial overlaps of consensus scopes are not depicted. Because the consensus scopes of clusters VII and XI are disjoint from all others, they are represented by isolated nodes.

Interestingly, many inclusion relations can be associated with a difference in the chemical elements within the metabolites. For example, the ATP consensus scope (cluster III) is completely contained in the consensus scope of APS (cluster XIII). ATP and all other metabolites of cluster III contain the elements C, H, O, N, and P. The four metabolites in the APS cluster contain additionally sulfur. This observation indicates that the chemical complexity plays an important role in the determination of the biosynthetic potential of a biochemical compound. However, there are examples in which metabolites possess a similar chemical composition and structure, but the corresponding consensus scopes differ greatly. For instance cluster IX, containing Acetyl-CoA, has a consensus scope being a small subset of the consensus scope of cluster XIII, containing APS. The members of both clusters are, however, composed of the same chemical elements (C,H,O,N,P,S). Thus, the obtained hierarchy of the metabolites according to their biosynthetic potential is not only determined by their chemical complexity.

### Combined Biosynthetic Potentials

So far, we have determined a hierarchy based on the biosynthetic potentials of single substances. However, a direct biological interpretation is hindered by the fact that it is unrealistic to assume that an organism will be provided with exactly one external substance. Usually, several nutrients are available and the exact composition may vary greatly for different organisms and different environments. To improve the biological significance of the developed concept, it is therefore of relevance to study the biosynthetic potentials of combinations of metabolites. Since a systematic analysis of seeds of a larger size is not feasible, we perform a Monte Carlo simulation and calculate the scopes for a large number of seeds consisting of a varying number of randomly chosen metabolites. We call the biosynthetic potential of a seed containing multiple compounds the *combined biosynthetic potential*.

The Monte Carlo approach is similar to that followed in [18]. There the authors also calculated a large number of combined scopes for randomly selected seeds. They studied the size distribution of the scopes and in particular the increase in scope size when systematically central metabolites such as ATP, NADH, Coenzyme-A or oxygen were added to the seed. Here, we address the question whether the identified combined biosynthetic potentials can unambiguously be assigned to the determined consensus scopes, thus confirming that the revealed hierarchical ordering is of a general nature.

For each seed size between 2 and 20, we generate 1000 random seeds and calculate the corresponding scopes. Based on the distance measure (Equation 1), we identify for each scope the most similar consensus scope and denote the similarity by *d*_0_. To assess the quality of the assignment to the closest consensus scope, we also identify the second nearest consensus scope and denote the distance by *d*_1_. The ratio *α* = *d*_0_/*d*_1_ quantifies the uncertainty of the assignment, with small values *α≪*1 reflecting unambiguous assignments and *α*≈1 indicating a large uncertainty, because in such a case the scope is equally similar to at least two consensus scopes.

**Figure 3:**
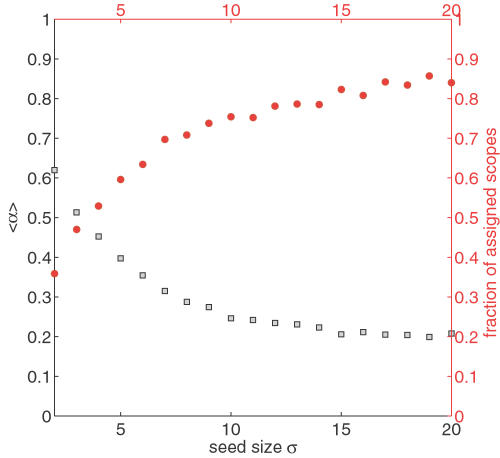
Uncertainty of cluster assignment and fraction of combined biosynthetic potentials assigned to a cluster. Shown are the average uncertainty *α* of the assignment to clusters (squares), and fraction of the combined biosynthetic potentials which have been assigned to one of the clusters I-XIII (circles) as a function of the seed size *σ*.

The average value *α* is plotted against the number of metabolites in the seed in Figure 3 (black squares). It can be seen that the assignment to a cluster is more reliable for larger seed sizes. This is not surprising since larger seeds tend to exhibit a larger biosynthetic potential and, as is the case for the potentials of single metabolites, small scopes cannot reliably be grouped into clusters. Consistent with the choice of parameters in the hierarchical clustering process, in which we merge two clusters if they exhibit a distance of less than 0.2, we only assign the scopes of multiple-compound seeds to a cluster if *d*_0_<0.2. As expected from the decreasing uncertainties of cluster assignment, the percentage of assigned clusters increases strongly with increasing seed size (red circles in Figure 3). For a seed size of two, less than 40% of the scopes are assigned to a cluster. For a seed size of 20, almost all scopes are unambiguously assigned to one of the thirteen clusters determined above.

**Figure 4:**
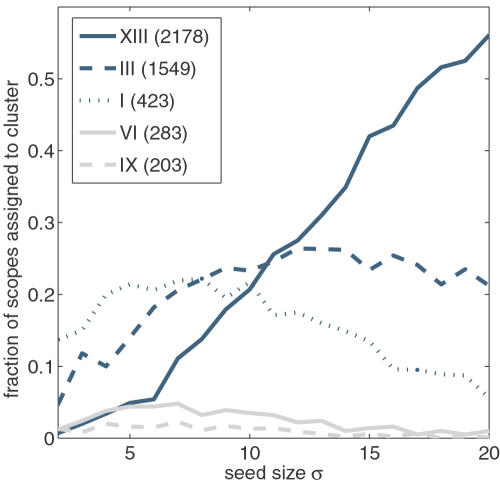
Assignment of combined biosynthetic potentials to selected clusters. The fraction of combined biosynthetic potentials, which are assigned to one of five selected clusters (XIII, APS; III, ATP; I, L-Glutamate; VI, dTTP; IX, Acetyl-CoA), is plotted versus the seed size.

To analyze which particular consensus scopes can be reached from a combination of metabolites, we plot in Figure 4 the fraction of scopes that are assigned to a particular consensus scope in dependence of the seed size. Shown are the values for the five clusters with the largest consensus scopes (XIII−APS, III−ATP, I−L-Glutamate, VI−dTTP, IX−Acetyl-CoA), all other clusters are assigned with negligible frequency. The frequency of assignment to the largest consensus scope increases strongly with increasing seed size. This is expected because the addition of new metabolites to the seed may only increase the biosynthetic potential, so that a randomly chosen large set of metabolites is more likely to display the full potential of metabolites from cluster XIII than a small set. However, the numbers provide further insight into the structural design of metabolism. For 20 randomly selected compounds, the chance that one of them belongs to the four compounds forming cluster XIII is still below 2%. On the other hand, more than half of the scopes for this seed size get assigned to the corresponding consensus scope. This indicates that the particular, chemically very rich, compounds from cluster XIII are not necessary to obtain the full biosynthetic potential characterized by the scope of APS. Instead, the same potential is contained in many combinations of smaller substances. This result generalizes the observation made in [14] that the scope of APS may also be reached if, for instance, CO_2_, NH_3_, phosphate, sulfate, water and oxygen are used as seed. The frequency of assignment to the second largest consensus scope also increases with increasing seed size, however, it does not change considerably for seed sizes larger than 10. For the assignment to the consensus scope of cluster I, and in fact for the other clusters as well, the frequency also increases for small seed sizes but tends to decrease when the seed sizes become large. The reason is that for larger sets of seed compounds it becomes increasingly difficult to find such combinations which do not exhibit a large biosynthetic potential. Therefore, for larger seed sizes, the frequency of assignment is shifted towards the larger consensus scopes.

These investigations demonstrate that the notion of consensus scopes is also meaningful in the context of combined biosynthetic potentials. Moreover, the larger the set of metabolites for which the combined biosynthetic potential is considered, the more combinations are assigned to one cluster. In fact, larger sets of metabolites tend to display a potential characterized by the largest consensus scopes. For large seed sizes (*σ*>20), this is true for more than half of all combinations. As a consequence, large sets of metabolites may not be distinguished by their combined potential, reflecting the fact that central metabolites such as nucleotide phosphates, amino acids and coenzymes may be built flexibly from many different resource combinations. We conclude that the hierarchical ordering of biosynthetic potentials which was determined for single metabolites is of a general nature and its significance even increases when combined biosynthetic potentials are studied.

## Discussion

The aim of this article is to identify organizational principles in metabolic networks. For this, we compared the biosynthetic potentials of biochemical compounds in the KEGG database. A potential is given by the set of all metabolites that can be produced from a compound. This set is called the scope of the compound [14]. We performed a hierarchical clustering to identify clusters of compounds with similar scopes. The analysis resulted in 12 clusters with a reasonable size (at least 10 metabolites) and one special cluster containing four metabolites with the largest biosynthetic potential. Within each cluster, the metabolites are chemically similar in the sense that from each of the cluster members a similar set of biochemical compounds can be produced. These findings inspired the definition of a *consensus scope* which describes an average set of metabolites which can in principle be produced from biochemical compounds found in one cluster. A detailed analysis of the compounds which are contained in each cluster showed that the similarity in biosynthetic potential can be related to particular chemical properties.

### Chemical Versus Functional Complexity

By calculation of the overlap of the consensus scopes we derived a hierarchy of metabolites, providing a novel view on the global organization of metabolism. Some compounds (as those of cluster III) possess a high chemical complexity in the sense that they are composed of many distinct functional groups that are essential for the synthesis of a large number of chemical compounds. Other metabolites possess a less complex chemical structure, and therefore the cellular metabolism can produce only a small number of compounds from them. For some of these cases, the lower biosynthetic potential can easily be explained by the presence or absence of chemical groups. For example, it is intuitive that from sugars (cluster V) less can be synthesized than from sugar phosphates (cluster IV).

In other cases, however, it is not immediately evident from the chemical structure alone why the biosynthetic potential of some metabolites is smaller than for others. For example, from deoxynucleotides with thymine as base (cluster VI), the metabolism can produce only a small subset from those which can be produced from deoxynucleotides with other bases (cluster III). This is surprising in view of the fact that thymine is structurally similar to, for example, uracil. In fact, the chemical structure of thymin is even slightly more complex since it possesses an additional methyl group. The different scope sizes of dTTP and dUTP result from the fact that dTTP is included in the scope of dUTP, whereas the opposite is not true. Because in our analysis all reactions are considered to be reversible, this asymmetry does not arise from thermodynamic constraints, but is rather an intrinsic structural property of the metabolic network. The issue of interconvertibility is discussed in detail in [14].

We hypothesize that the differences in scope size are a consequence of different biological functions of these compounds. In particular, synthesis of DNA in the presence of high levels of dUTP promotes incorporation of uracil into DNA, since polymerases cannot discriminate between the deoxynucleotides dUTP and dTTP [19]. Uracil misincorporation compromises the stability of DNA, resulting in DNA damage and cytotoxicity [20]. In normal cells, accumulation of dUTP by phosphorylation from dUMP via dUDP is avoided by rapid reductive methylation of dUMP to dTMP. Because a direct conversion of dTMP to dUMP is not possible, the concentration of dUMP is kept at a low level.

Our hypothesis concerning the asymmetry of interconvertibility of dTTP and dUTP is supported by experimental findings, in which the amoebozoa *Physarum Polycephalum* was grown on ^14^C-labeled nucleosides [21]. There, the authors observed that the ^14^C from thymidine only enters dTTP, whereas the ^14^C from other nucleosides was found in other ribonucleoside and deoxyribonucleoside triphosphates. In our analysis this is reflected by the presence of dTTP in the scopes of dATP, dGTP, dUTP, and dCTP, whereas the scope of dTTP does not contain any of these nucleoside triphosphates.

This example demonstrates that chemically very similar substances may differ significantly in their biosynthetic potentials and that these differences may only be explained by consideration of the biological functions of the metabolites. Apparently, the complexity of a chemical substance may be described in two different ways. The structural complexity of a metabolite is determined by the types and numbers of chemical groups and elements and the bonds between them. Another determinant is the oxidation number of a metabolite since oxidation/reduction reactions play an important role in metabolism. In contrast, the functional complexity of a metabolite is determined by its biological role within cellular metabolism. It may depend on the availability of appropriate enzymes, the subcellular and tissue-level localization of metabolites and enzymes, and the kinetics and thermodynamics of biochemical reactions. In this work, we invoked the concept of scopes, characterizing the biosynthetic potential of a metabolite, to provide a quantification for the functional complexity. Our results have indicated that both types of complexity are in many cases correlated, however, this correlation is not strict and we identified chemically very similar metabolites exhibiting a drastically different functional complexity. It will be interesting to study the relation between structural and functional complexity of metabolites in further detail.

### Primary and Secondary Metabolites

The analysis of the consensus scopes revealed a hierarchical setting in which some consensus scopes are contained in others. Two clusters are not included in this hierarchy, namely clusters VII and XI, which display a disjoint consensus scope from all others. Both clusters are composed of compounds with particular chemical features exhibiting a specific biological function. The compounds in cluster VII (eicosanoids), function as autocrine and paracrine mediators. Compounds in cluster XI, characterized by an indole group, act as signaling molecules in plants.

As a general tendency, we found that compounds belonging to clusters with a large consensus scopes are primary metabolites, i.e. metabolites necessary for essential cellular functions and present in the metabolism of most organisms. In contrast, compounds within clusters with a small biosynthetic potential are often secondary metabolites, for example alkaloids, terpenoids or fatty acids. Such metabolites are species specific and are not directly involved in essential cellular processes.

The chemical structure of many secondary metabolites is more complex than that of primary metabolites. Our investigations revealed that secondary metabolites within one cluster are structurally very similar and can be obtained from other members of the cluster through small chemical modifications such as methylation, hydroxylation or isomerization. In contrast, primary metabolites clustered in the same group often display large chemical differences. Despite this, the structure of the metabolic network ensures that such metabolites are still interconvertible, however, the pathway leading from one substrate to a product may require a large number of enzymatic reactions.

### Robustness and Universality of Metabolism Organization

In the first part of this work, we focused on the calculation of single scopes, i.e. sets of metabolites which can be produced if exactly one metabolite plus water and oxygen is available. We then asked whether our results are of a general nature and still hold true for combined scopes (sets of metabolites which can be produced from a larger number of initial substrates, i.e. a larger seed size). We applied a Monte-Carlo approach, randomly selecting seeds of varying sizes, and measured the distance between the scopes of the multiple-compound seeds to the consensus scopes of the clusters I-XIII. We found that the larger the seed sizes, the more combined biosynthetic potentials can be assigned to the 13 clusters previously identified. Since larger seeds tend to exhibit a larger biosynthetic potential, the frequency of assignment to the largest consensus scope (cluster XIII) increases strongly with increasing seed size. Thus, many combinations of smaller substances can exhibit the same biosynthetic potential as the chemically complex compounds from cluster XIII. This hints at a redundancy principle in the design of the global metabolic network. Our findings demonstrate that the hierarchical ordering of biosynthetic potentials, originally determined for single metabolites, is of a general nature, and also meaningful for larger sets of nutrient seeds.

Another study based on the Monte-Carlo approach [18] has shown that the sizes of the resulting scopes are concentrated in four disjoint regions, the largest scopes being produced only if oxygen is contained in the initial set of substrates. In the results presented above, we have always assumed that oxygen is present. We have tested whether our findings are dependent on this assumption and repeated our calculations for anaerobic conditions (see Methods, Effect of Oxygen; and Table S3 and Dataset S2 in the supporting information). In agreement with the results of Raymond and Segrè [18], we also observe that many metabolites possess a strongly reduced biosynthetic potential under anaerobic conditions, demonstrating that they can only deploy their full potential when oxygen is available. As a consequence, the clusters tend to be of a smaller size, but pairs of corresponding clusters can clearly be identified. In contrast, the consensus scopes, characterizing the typical biosynthetic potentials of the clusters, remain almost completely unchanged. It is remarkable that, while oxygen naturally has a strong impact on metabolism and possible synthesis routes, its absence or presence is not decisive for the hierarchical organization of the global metabolic network comprising enzymatic reactions from aerobic as well as anaerobic organisms. This fact supports the hypothesis that the computationally derived hierarchy is indeed of general nature and does not depend on specific assumptions.

Interestingly, clusters I, II and VIII which exhibit a considerable reduction under anaerobic conditions, have consensus scopes containing metabolites with a higher oxidation state than compared to consensus scopes of other clusters. Thus, many compounds contained in these consensus scopes are obtained from oxidation reactions, which in many cases require oxygen. The fact that many oxidation reactions take place using NAD^+^ or FAD as oxidant provides an explanation that the consensus scopes can still be reached, however by a smaller number of compounds. We expect that a more detailed investigation of the effect of the average oxidation number within clusters and their consensus scopes on the cluster reduction under anaerobic conditions will provide new insight into the role of oxygen and alternative oxidants in cellular metabolism.

Summarizing, by grouping metabolites with respect to their biosynthetic potential, the huge variability of biochemical compounds involved in metabolism can be represented in a relatively concise form. Apparently, there exist only a small number of typical sets of metabolites (the consensus scopes) which can be produced from one single precursor. These sets display a hierarchy which in some cases can be explained by the chemical groups contained in the precursors. In other cases, the underlying reasons for the hierarchical structuring have their origin in different biological functions of the compounds. Our results have been obtained from a computational study which is based on a database with necessarily incomplete and constantly changing content. Moreover, there is some degree of arbitrariness in the curation process used to derive the metabolic network. Despite these uncertainties, we could demonstrate that our results are only marginally different when based on database releases between which over two years have passed (see Methods, Robustness Against Changes in Network Structure; and Dataset S2 in the supporting information). More importantly, the derived hierarchical structure proved stable. We thus assume that the hierarchy is indeed an intrinsic characteristic of the metabolic network itself.

### Metabolism Hierarchy and Biological Evolution

The catalyzing enzymes are a product of a long evolutionary process which was governed by selection and mutation principles. In total, they catalyze only a small fraction of all theoretically possible chemical transformations. The nature of the evolutionary driving forces which resulted in the selection of the particular set of enzymatic reactions found in contemporary organisms remains subject to speculation. Our analyses show that the network is extremely flexible in its resource requirements, exhibited by the fact that central metabolites (e.g. ATP, NADH, Coenzyme-A, amino acids) may be synthesized from many different combinations of substrates. Nevertheless, certain metabolic routes involving chemically similar compounds are to some degree separated. This indicates that for the chemically feasible reactions that could provide the link, enzymes have not evolved. Of course, it is possible that such enzymes do exist but have not been discovered yet and are therefore not yet included in the KEGG database. However, database versions almost three years apart did show the same cluster separations. With the present rate of increase of biochemical knowledge, this is a hint that such enzymes do indeed not exist in contemporary organisms or are extremely rare.

Assuming that the observed separation of metabolism is a real feature of the contemporary metabolic network, what could have been a selective advantage that hindered the evolution of enzymes for connecting reactions? Of course, under physiological conditions many similar chemicals, also such displaying an identical scope, may exhibit different biological functions. This is possible by mechanisms such as allosteric regulation and gene regulation to adjust production rates to the present demand, so a structural separation of metabolic routes does not seem necessary from a present-day view. However, it can be assumed that early during metabolic evolution, primitive precursors of contemporary enzymes have catalyzed biological reactions. Common mechanistical themes in diverse enzyme superfamilies [22] suggest that early enzymes displayed a significantly lower substrate specificity, and the modular structure separating catalyzing from regulatory domains in proteins [23] allows to speculate that domains for functional control have been less elaborated or non-existent in early enzymes. In such a scenario, a separation of the metabolic network on the structural level does indeed make sense, since in this way certain chemical conversion routes are principally excluded, providing a selective advantage by avoiding undesired interactions. It is remarkable that the clearest separation involves nucleotide phosphates, which, as prerequisite for the genetic code, assumably have acquired a central role early during metabolic evolution. Moreover, the particularly similar nucleotides with strikingly different potentials, uracil and thymin, are exactly those structural elements which distinguish the related, information coding macromolecules DNA and RNA. We therefore hypothesize that the observed separation of clusters of similar compound is a relict of the early phase of metabolic evolution, when regulatory mechanisms had not yet evolved to their present-day elaboration. Under such conditions, it might have provided a selective advantage to fundamentally separate metabolic routes, which is most drastically achieved by a separation on a structural level.

One aspect poorly understood is how a large chemical diversity of more than 200.000 secondary species-specific metabolites has evolved from primary metabolic pathways [24]. The high plasticity of secondary metabolism involving enzymes with broad substrate specificity might have enabled organisms to adapt easily to environmental changes. A model has been proposed to explain the increased generation of chemical diversity after a mutational event assuming a broad substrate specificity of the enzymes [25]. In this model, a substrate A is sequentially converted by a series of distinct enzymes into other compounds B, C, D and E. A mutational event could give rise to a new variant of the organism, producing a compound A' that is structurally similar to A. The same enzymes acting on the pathway A→E could generate the new compounds B', C', D' and E'. Our results are consistent with such a model; secondary metabolites are confined to small clusters and the majority of these metabolites are interconvertible, being also found in the consensus scopes. Likely, most enzymes that might have catalyzed the transition from B to B' have not evolved, which explains the disjoint consensus scopes of these clusters. We expect that a clustering analysis of organism-specific networks may bring some insight in our understanding of the evolutionary transition from primary to secondary metabolism. We hypothesize that in early-evolved organisms some secondary metabolites or related compounds will be found in larger clusters functioning as primary metabolites.

While the presence or absence of oxygen is not influencing the hierarchical organization of the global network discussed here, we expect that the effect of oxygen on organism-specific hierarchies is more pronounced. Metabolism under aerobic and anaerobic conditions may differ considerably between organisms. For example, earlier organisms, which appeared before oxygen was present at a high concentration in the atmosphere, prefer fermentation (anaerobic) to oxidative phosphorylation (aerobic). We expect that i) the organism specific hierarchies will reflect the growth conditions (aerobic versus anaerobic) and ii) the effect of oxygen will stronger influence the structure of the metabolism for aerobic than for anaerobic organisms. From such comparative studies we expect to gain further clues about which underlying principles may have guided the evolution of metabolic networks.

## Methods

### Data Import

We have retrieved the global metabolic network from the KEGG database in the following way. From the LIGAND subdivision, the complete list of reactions has been imported. The reactions have been checked for consistency and those were rejected which showed an erroneous stoichiometry, by which we mean that some atomic species occurred in different numbers on both sides of the reaction. The inclusion of such erroneous reactions could result in absurd events such as the creation of chemical elements or groups. We identified compounds possessing ambiguous structure information, such as chains of chemical groups of unspecified length (e.g. Ubiquinol, KEGGID: C00390, C14H20O4[C5H8]n) or compounds with unspecified residues (e.g. Amino acid, KEGGID: C00045, C2H4NO2R). We rejected all reactions involving such metabolites. Further, we did not include reactions involved in glycan synthesis because the focus of our investigation lies on the metabolism of small chemical species, which also does not include the formation of complex structures such as proteins or RNA and DNA molecules.

This curation process has been applied to two different database releases, one dating back to January 2005, and a recent version from December 2007. The older release resulted in a network comprising a set of 4811 enzymatic reactions connecting 4104 biochemical compounds. Results presented in the main text have been obtained for this network. To ensure that our results are not critically influenced due to changes in the database, calculations have been repeated for the network derived from the recent database release, comprising 5529 reactions with 4668 reactants (see below).

We have also tested whether the curation process applied to extract the metabolic network from the database is critical for our results. For this purpose, we built two more networks from the recent database version, one with relaxed and one with stringent criteria. For the former, all 6003 reactions were included, even those showing erroneous stoichiometry. For the latter we demanded absolute correctness, risking the exclusion of valid reactions, leaving 4257 reactions in the network. For all networks, the complete reaction lists are provided in the supporting information (Dataset S2).

It is possible that by removing reactions during curation the resulting network contains parts not connected to the rest of the network. This was indeed observed; however, in most cases this concerns single or groups of a small number of reactions. We did not put any effort in deriving a fully connected network, since small disconnected components are unproblematic for the kind of analysis presented here.

In principle, the KEGG database also provides information on the reversibility of biochemical reactions. This information is contained in XML files which define the organism-specific pathways. We found, however, that for many reactions (over 200), this information is ambiguous. Further, the direction in which a reaction actually proceeds under physiological conditions is strongly dependent on the metabolite concentrations and therefore may vary for different organisms, tissues or environmental conditions. To account for this and considering that in principle every enzymatic reaction may also proceed in the reverse direction, we have considered all reactions to be reversible.

### Network Expansion and Scope Calculation

To assess the synthesizing capacities of a metabolic network when it is provided with a particular substrate, we apply the method of network expansion which is in detail described in [14]. We give here a short outline of the algorithm:

1. A particular metabolite, *X*, for which the scope shall be calculated, is selected. We define a set *M* = {*X*, H_2_O, O_2_} containing this metabolite, water, and oxygen.
2. All reactions within the metabolic network are identified for which all substrates are present in the set *M*. These reactions can in principle operate when the metabolites contained in *M* are present.
3. For each of the reactions identified in the previous step, the products are added to the set *M*, leading to an expanded set of metabolites.
4. Steps 2 and 3 are repeated until in step 3 no new metabolite can be added to the set *M*.

The resulting set *M* is called the *scope of compound X*. We use the scope as a measure for the biosynthetic potential carried by metabolite *X*. Naturally, this algorithm can be initialized with any combination of seed compounds. For the analysis of combined biosynthetic potentials, we randomly select several metabolites as seeds and apply the described algorithm.

### Hierarchical Clustering

We apply a hierarchical clustering with the distance measure (Equation 1). The advantage of hierarchical clustering methods is that they provide information about clustering of the data at all scales, from fine to coarse. The disadvantage is the computation time which scales with *O*(*n* ^2^), because the distance between every pair of data has to be computed.

We choose a nearest neighbor group-average clustering algorithm [26]. Nearest neighbor clustering is a bottom up clustering method where iteratively the elements or clusters with the smallest distance are joined. Group-averaging refers to the method of defining the distance between two clusters as the average over all distances between pairs of the corresponding cluster elements. We denote the elements (scopes) of cluster *i* by 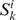 and of cluster *j* by 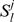, and the sizes of clusters *i* and *j* by *s^i^* and *s^j^* respectively. The distance *d_ij_* between the two clusters *i* and *j* is then defined as the group-average of the distances between all elements 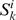 and 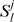, for *k* = 1, 2,…*s^i^* and *l* = 1, 2,…*s^j^*,
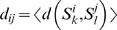
(2)where the average is over all *k* and *l*. The algorithm is implemented as follows:

1. The distances between all pairs of scopes are computed and stored in a matrix *DistSc*. To every data point we assign a cluster label, i.e. initially every data point is itself a cluster.
2. In the first step we find the smallest element of the matrix *DistSc*(*i*, *j*) = min(*DistSc*) and assign the same cluster label to both of them, say *m*.
3. For the group-averaging, we then modify the matrix *DistSc* by removing one row and column (say *i*). Row *j* and column *j* are replaced by the distances between the newly formed cluster *m*, consisting of the former scopes *i* and *j*, to the remaining clusters, according to Equation 2.
4. Steps 2 and 3 are then repeated until all scopes are merged into the same cluster. Furthermore, for every iteration we store the current minimum element of *DistSc*.

**Figure 5:**
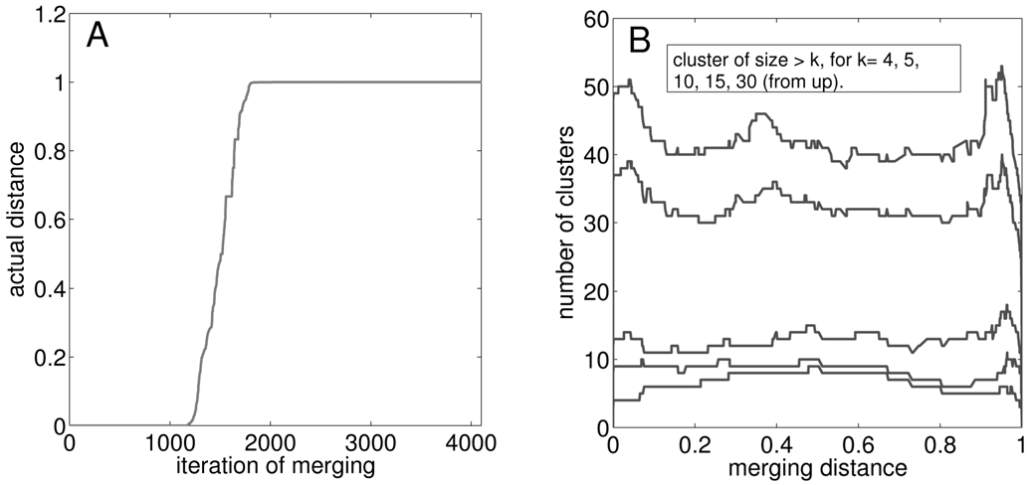
Hierarchical clustering method: choice of clustering level. (A) Increasing distances at which clusters are merged at successive iterations of the nearest neighbors clustering. (B) Number of clusters of a given minimum size versus the joining distance at each iteration.

The result obtained in this procedure is a clustering of the data on various scales. At the first iterations only very similar elements obtain the same cluster label and the clustering is very fine. Towards the end elements or clusters with large distances are joint, resulting in a coarse clustering with a smaller number of clusters. Figure 5A shows the increasing distances at which elements or clusters are merged at subsequent iterations of the nearest neighbors clustering. In the beginning elements are clustered at very small distances, in fact there is quite a large number of identical scopes. In the next region the distances increase linearly to the maximum value. The following iterations then join elements that do not have even a single common substance in their scopes. All the very small scopes of compounds with zero synthesizing capacity are assigned to clusters in this last phase.

After the clustering is finished, the next step is to decide which scale of the clustering is reasonable for analysis. Our choice is based on the distances themselves on one hand, and on the robustness of the clusters on the other. Naturally, elements of the same cluster should be very similar, therefore the coarse scale, where clusters are joined although the distance between them is large (let's say, *d_ml_*>0.5) is not feasible.

The robustness of the clusters is measured as the variation in the amount of larger clusters when varying the joining distances. In Figure 5B we plot the number of clusters of a given minimum size *k* for various *k* as a function of the distance at the current iteration of clustering. The length of a plateau in one of the curves then gives the robustness of the clustering for clusters of size larger than *k*. We are interested in larger clusters and therefore consider only the three lower curves. There is a smaller plateau for distance values between 0.1–0.2 and a long one between 0.3–0.7. Because of the requirement that the distances should be rather small, we focus on the first plateau, and therefore choose the clustering level where elements are joint with a distance of at most 0.2.

**Figure 6:**
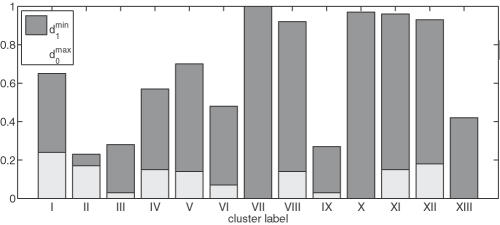
Cluster radius and cluster separation. Maximum distance between compounds of a cluster to their corresponding consensus scope *d*_0_ (cluster radius), and the minimum distance of the compounds to the second nearest consensus scope *d*_1_ (cluster separation). The assignment to a cluster is good if *d*_0_≪*d*_1_.

Finally, we measure the quality of the clustering to assure that the elements within the same cluster are similar and the clusters well distinguishable. Generally, a clustering is considered good, if the distances of the elements within a cluster are small, and the distances between elements of distinct clusters large. To quantify the quality of the clustering we compute for every cluster (I-XIII) the distance between all scopes contained in the cluster to the consensus scope. The maximum of these distances can be regarded as a *cluster radius*, denoted by 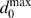. Furthermore, we compute the distance between all scopes in the cluster to the second nearest consensus scope. The minimum of these, 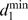, provides a measure of the *cluster separation*. Since the distance is based on the Jaccard coefficient, 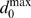 and 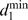
≤1. A cluster is well defined if 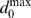 is small and 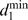 large. Figure 6 shows that for all clusters 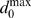 is much smaller than 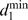, except for cluster II. This can be regarded as a consequence of the similarity between the clusters II and IX. Cluster II is fully embedded in cluster IX, while their consensus scopes differ only by about 25%.

To obtain a single parameter quantifying the *uncertainty of the assignment* of metabolites to a cluster, we compute the ratio 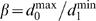. The quality of the clustering is good for *β*≪1. For *β*≈1, the assignment is uncertain, as in this case the scope is equally similar to at least two consensus scopes.

### Robustness Against Changes in Network Structure

The results presented in this work have been obtained for a metabolic network reconstructed from a KEGG database version dating back to the year 2005. Due to our rapidly increasing biochemical knowledge, it lies in the nature of databases as KEGG that their content is constantly changing. It is therefore a crucial question whether our results, and therefore our biological conclusions presented here, are still valid if new reactions enter the database or erroneous reactions are removed. An indication that the results should not be drastically influenced is given by the robustness studies of single scopes in [14]. Here it was shown that deletions of single reactions from the network alter the scopes only in rare cases considerably.

Since it is unclear how the clusters and the consensus scopes are affected when the network structure is altered by several hundreds of reactions, we have repeated our calculations for a network derived using a database version from December 2007 (for the detailed results, see Dataset S2 in the supporting information). For the new network we compared the obtained clusters, consensus scopes and resulting hierarchies to the results above.

For all the clusters I−XIII, corresponding clusters can easily be identified for the new network (see Tables S1 and S2, and Figure S1). The similarity between the clusters I−XIII and the corresponding new clusters is very high. The Jaccard coefficients of pairs of corresponding clusters have in most cases values above 0.7. The corresponding consensus scopes are even more similar, Jaccard coefficients of pairs of corresponding consensus scopes have values of 0.8 or higher. As expected from the increase in network size, the cluster sizes as well as the consensus scopes have a tendency to increase in size. Apart from identifying corresponding clusters, we also obtained new clusters from the recent database version. Two clusters are formed by carotenes and oxylipins derived from linolenic acid, containing 18 and 10 metabolites, respectively. Their consensus scopes (sizes 28 and 20, respectively) do not overlap with other consensus scopes. Again we see that compounds contained in isolated clusters are secondary metabolites.

The effect of the specific network curation strategy has been tested with two more networks derived from the recent KEGG database version (see above), termed relaxed and stringent. Not surprisingly, we found that for the relaxed network our results change considerably. Erroneous stoichiometries lead to absurd events, like the creation of new chemical elements. One example is that for this network the scopes of ATP and APS are identical even though APS possesses a sulfate group while ATP does not. Due to such obviously meaningless results, we do not consider the relaxed network further. However, such observations demonstrate how important the process of database curation is to derive consistent network models. For the stringent network the scope sizes–and therefore consensus scope sizes–are sometimes drastically reduced, however, most of the previously identified clusters are again present, and the obtained hierarchy is still structurally conserved (see Figure S3). The major exception is that cluster III, represented by ATP, splits into two clusters exhibiting consensus scopes identical to the scopes of ATP and UTP, respectively. Moreover, some of the smaller clusters disappear. We explain these changes by the fact that some important reactions are missing due to the very harsh criteria. The detailed results can be found in the supporting information (Dataset S2).

### Effect of Oxygen on the Hierarchical Structure

We have performed our calculation assuming that oxygen is always available. This assumption is motivated by the fact that atmospheric oxygen, a highly reactive chemical, has been abundant for approximately 2.8 billion years and therefore the metabolic network that we see today has to a large extent evolved under aerobic conditions. However, there is a certain arbitrariness in our assumption, since for similar reasons other compounds, such as CO_2_, could be included in the seed. To verify whether our calculations critically depend on the availability of oxygen, we repeated the cluster analysis based on biosynthetic potentials of all metabolites under the premise that only water is additionally available. Corresponding clusters with a high overlap can easily be identified between the results obtained with and without oxygen. The results are summarized in Tables S2 and S3, details are found in Dataset S2 and Figures S2 and S4. Some clusters remain completely unchanged (IV, X, and XIII), whereas others are slightly reduced in size. Interestingly, while absence of oxygen does alter the cluster composition, most consensus scopes remain completely identical. A significant change in size is observed for cluster VII, which is almost halved in the absence of oxygen, while the corresponding consensus scope is unaffected. A closer inspection reveals that this cluster is in fact split into two subclusters, a smaller one formed by prostaglandins and thromboxanes whose consensus scope is completely contained in that of the larger subcluster formed by lipid hydroperoxides, leukotrienes and oxilins. The reduced biosynthetic capacity of prostaglandins and thromboxanes is due to an oxidation reaction of prostaglandin H2 yielding prostaglandin G2, which does not occur under anaerobic conditions. The most dramatic change is observed for cluster VIII, which collapses completely. The metabolites within this cluster are very diverse but possess the common property that they can be oxidized to CO, CO_2_ or small carboxylic acids. The collapse of this cluster can be accounted to the fact that these oxidizing reactions cannot take place in the absence of oxygen, which also explains the strong reduction of the size of the corresponding consensus scope.

## Supporting Information

Figure S1 Hierarchy of metabolites for the network derived from the recent KEGG version.
(0.03 MB PDF)

Figure S2 Hierarchy of metabolites for the network derived from the recent KEGG version under anaerobic conditions (no oxygen in the seed).
(0.02 MB PDF)

Figure S3 Hierarchy of metabolites for the network derived with stringent curation strategy.
(0.02 MB PDF)

Figure S4 Hierarchy of metabolites for the network derived with stringent curation strategy under anaerobic conditions.
(0.02 MB PDF)

Table S1 Mapping from the cluster labels I-XIII to the identifiers of the corresponding clusters found in the recent network, both for aerobic and anaerobic conditions (directories ‘semiStrict’ and ‘semiStrict_no_o2’ in Dataset S2).
(0.01 MB PDF)

Table S2 Comparison of the clustering results for networks derived from two different versions of the KEGG database.
(0.01 MB PDF)

Table S3 The effect of oxygen on the clustering results and the consensus scopes.
(0.01 MB PDF)

Dataset S1 Definition, clusters, and consensus scopes of the network discussed in the text. The metabolic network which was retrieved from the KEGG database and subsequently curated is available as a list of KEGG reaction IDs (file ‘reaction_list.txt’). A full list of the hierarchical clusters (‘hierarchical_clusters.txt’) is provided along with a list of all corresponding consensus scopes (‘consensus_scopes.txt’). In total, 350 clusters and their consensus scopes are presented; clusters 1–12 correspond to clusters I-XII in the text, and cluster 63 is cluster XIII.
(0.33 MB TAR)

Dataset S2 Results for three networks obtained with different curation strategies. The dataset consists of five subdirectories, each containing three files corresponding to Dataset S1. All networks have been extracted from a recent KEGG database version as of December 2007. The three networks were obtained using very relaxed criteria for the exclusion of reactions (directory ‘relaxed’), identical criteria as was used for Dataset S1 (‘semiStrict’) and extremely stringent criteria (‘strict’). For the latter two, also results for anaerobic conditions are presented (‘semiStrict_no_o2’ and ‘strict_no_o2’).
(1.08 MB TAR)
