# Supplementary material for: Biosynthetic Potentials of Metabolites and Their Hierarchical Organization
Source: PLoS Comput Biol. 2008 Apr 4;4(4):e1000049. doi: 10.1371/journal.pcbi.1000049 (PMC2289774; doi:10.1371/journal.pcbi.1000049)
Supplement: Figure S1 — Hierarchy of metabolites for the network derived from the recent KEGG version. (0.03 MB PDF) [file pcbi.1000049.s001.pdf]

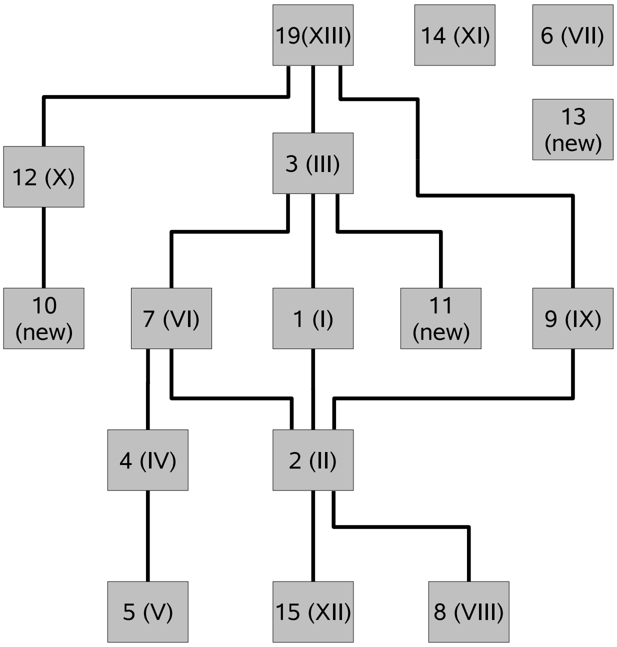

Figure S1: **Hierarchy of metabolites for aerobic conditions.** Same as Figure 2 in the main text, but calculated from a network derived from a recent KEGG database version from December 2007. To obtain the network identical curation criteria were used as for the network discussed in the text. The resulting network contains 5529 reactions connecting 4668 metabolites. Water and oxygen are present in all seeds. Cluster labels are given according to the supplementary files. In brackets corresponding clusters from the text are marked in roman numbers. It can be seen that some new clusters have been identified. This is not surprising in view of the fact that the database is increasing in size since new enzymes are continuously discovered. Interestingly, when restricted to those clusters which have been observed also in the previous network, the hierarchy is almost identical to the one obtained previously (compare to Figure 2 in the main text). The only difference is that the consensus scope of cluster X is no longer included in that of cluster III. The considerable increase in the size of the consensus scope (but not of the cluster) indicates that in the new version of the database some important reactions have been included which allow for the production of a special group of metabolites which are not contained in the consensus scope of ATP (cluster III).
