# Supplementary material for: Biosynthetic Potentials of Metabolites and Their Hierarchical Organization
Source: PLoS Comput Biol. 2008 Apr 4;4(4):e1000049. doi: 10.1371/journal.pcbi.1000049 (PMC2289774; doi:10.1371/journal.pcbi.1000049)
Supplement: Figure S2 — Hierarchy of metabolites for the network derived from the recent KEGG version under anaerobic conditions (no oxygen in the seed). (0.02 MB PDF) [file pcbi.1000049.s002.pdf]

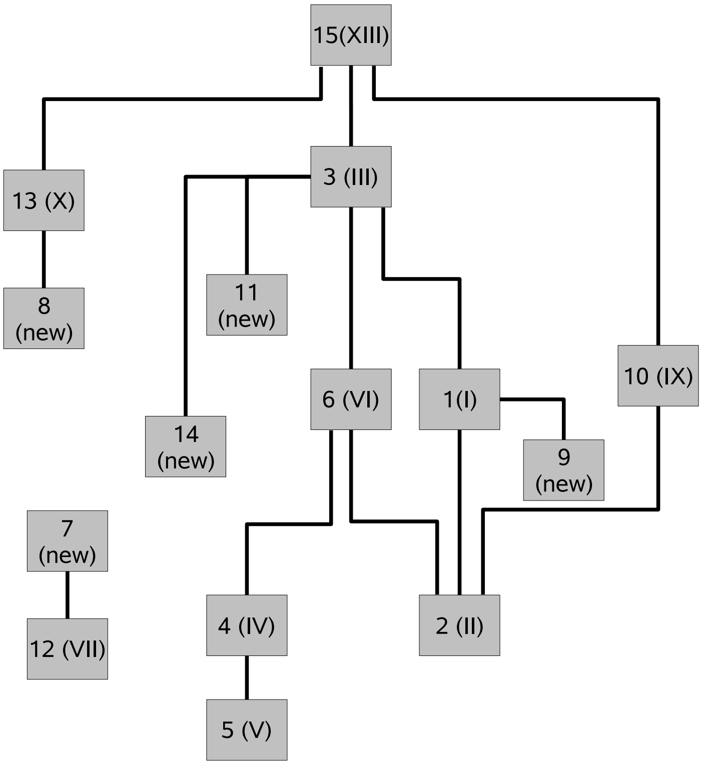

Figure S2: **Hierarchy of metabolites for anaerobic conditions.** Same as Figure S1, but only water (and no oxygen) has been added to the seeds. As discussed in the text, some clusters are reduced in size. Therefore, some of the small clusters are not included in the figure, whereas others are newly incorporated. It can be seen that the clusters also present in Fig. S1 are ordered in an analogous hierarchy as in the aerobic case.
