# Supplementary material for: Biosynthetic Potentials of Metabolites and Their Hierarchical Organization
Source: PLoS Comput Biol. 2008 Apr 4;4(4):e1000049. doi: 10.1371/journal.pcbi.1000049 (PMC2289774; doi:10.1371/journal.pcbi.1000049)
Supplement: Figure S3 — Hierarchy of metabolites for the network derived with stringent curation strategy. (0.02 MB PDF) [file pcbi.1000049.s003.pdf]

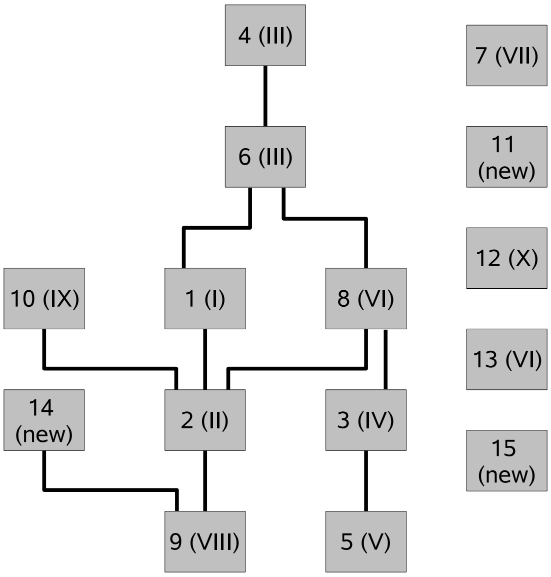

Figure S3: **Hierarchy of metabolites for aerobic conditions for a network curated with strict criteria.** Same as Figure 2 in the main text, but calculated from a network derived from a recent KEGG database version from December 2007 with a very stringent curation strategy (see section 4.1). The network contains 4257 reactions connecting 3782 metabolites. Water and oxygen are present in all seeds. At first glance, the hierarchy appears different from those diplayed in Figs. S1 and S2. However, this is mainly caused by the absence of cluster XIII and the fact that cluster III is now separated into two smaller clusters. As discussed in the text, we see the reason for the disappearance and the splitting of clusters in too strict criteria during curation. Probably, valid reactions have been excluded so that important connecting metabolic pathways are no longer present in the network. Interestingly, though, most clusters (I, II, III, IV, V, VI, VIII and IX) are ordered in an analogous way as shown in Figs. S1 and S2.
