# Supplementary material for: Biosynthetic Potentials of Metabolites and Their Hierarchical Organization
Source: PLoS Comput Biol. 2008 Apr 4;4(4):e1000049. doi: 10.1371/journal.pcbi.1000049 (PMC2289774; doi:10.1371/journal.pcbi.1000049)
Supplement: Figure S4 — Hierarchy of metabolites for the network derived with stringent curation strategy under anaerobic conditions. (0.02 MB PDF) [file pcbi.1000049.s004.pdf]

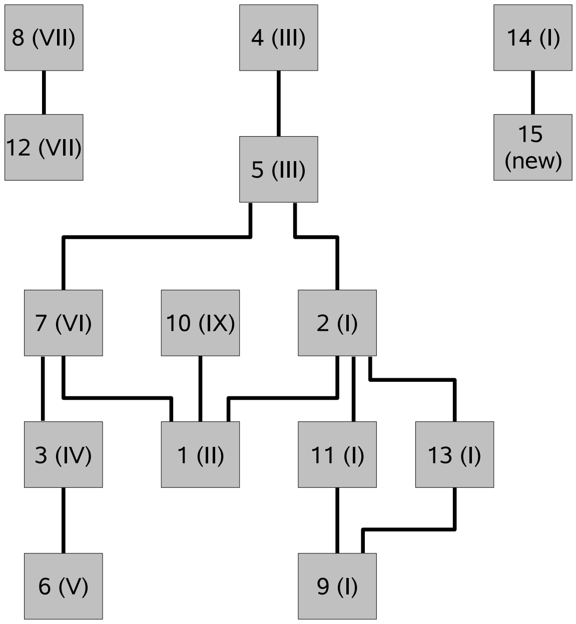

Figure S4: **Hierarchy of metabolites for anaerobic conditions for a network curated with strict criteria.** Same as Figure S3, but only water (and no oxygen) has been added to the seeds. Here, an interesting effect can be observed: Former cluster I splits into a number of subclusters, indicating that the effect of oxygen is more pronounced for this network containing only a limited set of reactions. A possible explanation is that important oxidizing / reducing reactions using other oxidants than oxygen are not included. Remarkably, the remaining clusters are still ordered analogously to the hierarchies diplayed in Figs. S1–S3.
