# Supplementary material for: Biosynthetic Potentials of Metabolites and Their Hierarchical Organization
Source: PLoS Comput Biol. 2008 Apr 4;4(4):e1000049. doi: 10.1371/journal.pcbi.1000049 (PMC2289774; doi:10.1371/journal.pcbi.1000049)
Supplement: Table S1 — Mapping from the cluster labels I-XIII to the identifiers of the corresponding clusters found in the recent network, both for aerobic and anaerobic conditions (directories ‘semiStrict’ and ‘semiStrict_no_o2’ in Dataset S2). (0.01 MB PDF) [file pcbi.1000049.s005.pdf]

Table S1: **Mapping of the cluster identifiers I–XIII used in the text to the identifiers found in the supplementary data files.** The internal numbering of the clusters is automatically done by the cluster identification algorithm which sorts the identified clusters by decreasing size. Therefore, same numbers do not necessarily stand for corresponding clusters of different scenarios. This table gives the cluster numbers which correspond to the thirteen clusters I–XIII which are discussed in the text.

Label	dataset S1	S2 (`semiStrict`)	S2 (`semiStrict_no_o2`)
I	1	1	1
II	2	2	2
III	3	3	3
IV	4	4	4
V	5	5	5
VI	6	7	6
VII	7	6	7
VIII	8	8	31
IX	9	9	10
X	10	12	13
XI	11	14	18
XII	12	15	19
XIII	63	19	15
