# Supplementary material for: Biosynthetic Potentials of Metabolites and Their Hierarchical Organization
Source: PLoS Comput Biol. 2008 Apr 4;4(4):e1000049. doi: 10.1371/journal.pcbi.1000049 (PMC2289774; doi:10.1371/journal.pcbi.1000049)
Supplement: Table S2 — Comparison of the clustering results for networks derived from two different versions of the KEGG database. (0.01 MB PDF) [file pcbi.1000049.s006.pdf]

Table S2: **Comparison of the clustering results for networks derived from two different versions of the KEGG database.** 'Old' refers to a database version from January 2005, 'new' refers to a version from December 2007. In the upper table the cluster sizes are given, the overlaps between corresponding clusters as well as the Jaccard coefficient quantifying the similarity. The lower table holds corresponding information on the consensus scopes characterizing the clusters.

Clusters

Label	old size	new size	overlap	Jaccard coeffient
I	261	306	248	0.78
II	183	217	168	0.72
III	102	103	100	0.95
IV	57	59	54	0.87
V	41	43	36	0.75
VI	34	25	19	0.48
VII	23	34	21	0.58
VIII	22	22	20	0.83
IX	19	19	19	1
X	13	12	11	0.79
XI	12	10	10	0.83
XII	10	10	10	1
XIII	4	9	4	0.44

Consensus scopes

Label	old size	new size	overlap	Jaccard coeffient
I	428	452	399	0.83
II	153	153	145	0.90
III	1554	1524	1351	0.78
IV	114	117	111	0.93
V	36	35	35	0.97
VI	310	293	275	0.84
VII	28	39	26	0.63
VIII	17	17	17	1
IX	208	215	199	0.89
X	54	86	49	0.54
XI	16	15	15	0.94
XII	14	14	14	1
XIII	2183	2445	2029	0.78
