# Supplementary material for: Biosynthetic Potentials of Metabolites and Their Hierarchical Organization
Source: PLoS Comput Biol. 2008 Apr 4;4(4):e1000049. doi: 10.1371/journal.pcbi.1000049 (PMC2289774; doi:10.1371/journal.pcbi.1000049)
Supplement: Table S3 — The effect of oxygen on the clustering results and the consensus scopes. (0.01 MB PDF) [file pcbi.1000049.s007.pdf]

Table S3: **The effect of oxygen on the clustering results and the consensus scopes.** Shown are the sizes of the clusters (upper part) and of the consensus scopes (lower part), as determined with and without oxygen in the seed. Also shown are the overlaps and Jaccard coefficients of corresponding sets. Both results have been derived for the network extracted from the newest version of the KEGG database (December 2007).

Clusters

Label	with oxygen	without oxygen	overlap	Jaccard coeffient
I	306	227	227	0.74
II	217	158	158	0.72
III	103	100	100	0.97
IV	59	59	53	0.82
V	43	32	31	0.70
VI	25	22	22	0.88
VII	34	19	19	0.56
VIII	22	5	5	0.23
IX	19	15	15	0.79
X	12	12	12	1
XI	10	8	8	0.8
XII	10	8	8	0.8
XIII	9	9	9	1

Consensus scopes

Label	with oxygen	without oxygen	overlap	Jaccard coeffient
I	452	452	452	1
II	153	153	153	1
III	1524	1524	1524	1
IV	117	117	117	1
V	35	26	26	0.74
VI	293	293	293	1
VII	39	39	39	1
VIII	17	9	9	0.53
IX	215	215	215	1
X	86	83	83	0.97
XI	15	12	12	0.8
XII	14	14	14	1
XIII	2445	2445	2445	1
